# Novel approach to sacroiliac joint pain: Preliminary cadaveric and in-silico analysis of safety and feasibility of X-ray-guided high-intensity focused ultrasound ablation

**DOI:** 10.1016/j.inpm.2026.100767

**Published:** 2026-05-19

**Authors:** Eric Miller, Michael Gofeld, Brian Skoglind, Arik Hananel, Suzanne Leblang, Jean-Francois Aubry, Lynn Kohan

**Affiliations:** a FUSMobile, 6120 Windward Pkwy, STE 210, Alpharetta, GA, 30005, USA; b Unika Medical Centre, 240 Duncan Mill Road Unit 800, North York, M3B 3S6, Canada; c Focused Ultrasound Foundation, 1230 Cedars Court Suite 206, Charlottesville, VA, USA, 22903; d Physics for Medicine Paris, INSERM U1273, CNRS UMR 8063, ESPCI Paris, PSL University, 10 rue d'Oradour-sur-Glane, 75015, PARIS, France; e University of Virginia, 545 Ray C. Hunt Drive, Charlottesville, VA, 22903, USA

**Keywords:** Sacroiliitis, High-intensity focused ultrasound ablation, Chronic pain, Back pain, Sacroiliac joint, Peripheral nerve ablation

## Abstract

**Background:**

Current treatment modalities for chronic sacroiliac joint (SIJ) pain each have advantages and limitations. High-intensity focused ultrasound (HIFU) is a novel, non-invasive tool which produces highly localized thermal ablation using common imaging platforms such as magnetic resonance imaging (MRI), ultrasound, and fluoroscopy. Regulators have approved Fluoroscopy- or X-ray-guided HIFU (XRgHIFU) in the USA, EU, UK, and Canada.

**Objectives:**

This study evaluates XRgHIFU's initial safety and feasibility as a non-invasive alternative to RFA for chronic SIJ pain.

**Methods:**

In a cadaveric model, thermocouples were implanted in targets along the lateral sacral crest and 1 cm into the foramen. The targets were then sonicated and temperature was recorded.

In an *in-silico* model, 10 adult computed tomography scans were simulated using a 3D, *k-Wave*-based acoustic and thermal simulation pipeline. Target and foraminal temperatures corresponding to the cadaveric model were recorded, and the resultant lesions were analyzed.

**Results:**

The cadaveric and *in-silico* models showed agreement. Neither model indicated ablation of the intra-foraminal nerve root. The ratio of heating at target vs. foraminal test point was 15.7 ± 4.6 (N = 6) in the cadaveric model, whereas the ratio in the *in-silico* model was 17.3 ± 9.3 (N = 20). Contiguous strip lesions ≥15 mm in width were produced in the *in-silico* model.

**Conclusion:**

This study supports the safety and feasibility of XRgHIFU for SIJ pain.

## Highlights

• Previously demonstrated: x-ray guided focused ultrasound safely ablates lumbar medial branch nerves.
• Focused ultrasound principles apply to sacroiliac joint pain treatment.
• Study measures heat exposure near spinal nerve roots during treatment.
• No harmful heating detected in critical nerve root structures.
• Results support future research using focused ultrasound for sacroiliac pain.

## 1 Introduction

Sacroiliac joint (SIJ) pain is a syndrome affecting the joint connecting the sacrum and pelvis that affects 15% to 25% of patients with axial low back pain [1], which itself has a 65% - 80% lifetime prevalence in adults in the United States [2]. For cases of chronic SIJ pain that last more than 6 months and are unresolved by conservative management, the standard of care is therapeutic SIJ injections or diagnostic posterior sacral lateral branch nerve blocks, followed by radiofrequency ablation (RFA) and/or SIJ fusion [1].

RFA for SIJ pain most often targets the L5 dorsal ramus, S1-S3 dorsal rami lateral branches, and sometimes includes the L4 and/or S4 dorsal rami, depending on patient anatomy and diagnostic block results [3]. This procedure is time-consuming, requiring insertion of multiple RFA cannulas or a customized RFA device. Despite strong evidence supporting the efficacy of sacral lateral branch RFA [[4], [5], [6], [7]], its use in the United States for SIJ pain has sharply declined since the Medicare Administrative Contractors rescinded coverage for lateral sacral branch RFA (CPT Code 64625) in 2023 [8].

In patients with persistent pain despite RFA, SIJ fusion is a minimally invasive surgery which reduces pain but results in decreased mobility and requires significant recovery time [[9], [10], [11], [12], [13]]. SIJ pain can also occur when a lumbar fusion is followed by adjacent segment disease. Given the lack of coverage for RFA in the United States, few treatment options exist for many patients with SIJ pain [[9], [10], [11], [12], [13]].

High-intensity focused ultrasound (HIFU) is an emerging technology to treat a variety of painful conditions [[14], [15], [16], [17]]. The United States Food and Drug Administration (FDA), *Conformité Européenne* (CE), and Health Canada have already approved fluoroscopy- or X-ray-guided HIFU (XRgHIFU) to treat facetogenic lumbar pain. Most publications to date have used MRI-guided HIFU (MRgHIFU) for SIJ treatments [[14], [15], [16], [17]], which involves complex imaging and anatomic considerations and is costly [18]. Bulat et al. demonstrated that a continuous lesion can be created on the sacral lateral branch nerves with a handheld HIFU device in a swine model [18]. XRgHIFU for treatment of SIJ pain has yet to be extensively studied, despite publication of initial clinical and preclinical data for HIFU for facetogenic pain [[19], [20], [21]], a procedure technically similar to SIJ XRgHIFU treatment. XRgHIFU has previously been and is currently being used in clinical trials for SIJ pain [22,23] and has the advantages of faster targeting and lower cost than magnetic resonance-guided focused ultrasound.

In this study, we quantified safety considerations for XRgHIFU for SIJ pain using the Neurolyser XR device and analyzed the feasibility of this treatment with *in-silico* and cadaveric models. Initial safety and targeting considerations were analyzed using *k-Wave* and computed tomography (CT)-based simulation models from 12 CT datasets and a relative thermometry cadaveric model. These models were compared and analyzed independently to quantify intra-foraminal heating due to the XRgHIFU lesion and document the location, size, and continuity of the expected thermal lesion.

## 2 Methods

### 2.1 Cadaveric studies

Cadaveric studies were performed at the University of Virginia's Surgical Skills Training Center (Charlottesville, VA, USA) on two cadavers embalmed using a “Soft Cure Embalming” method [24]. This method preserves the acoustic properties of cadaver tissue better than embalming using formalin.

**Fig. 1:**
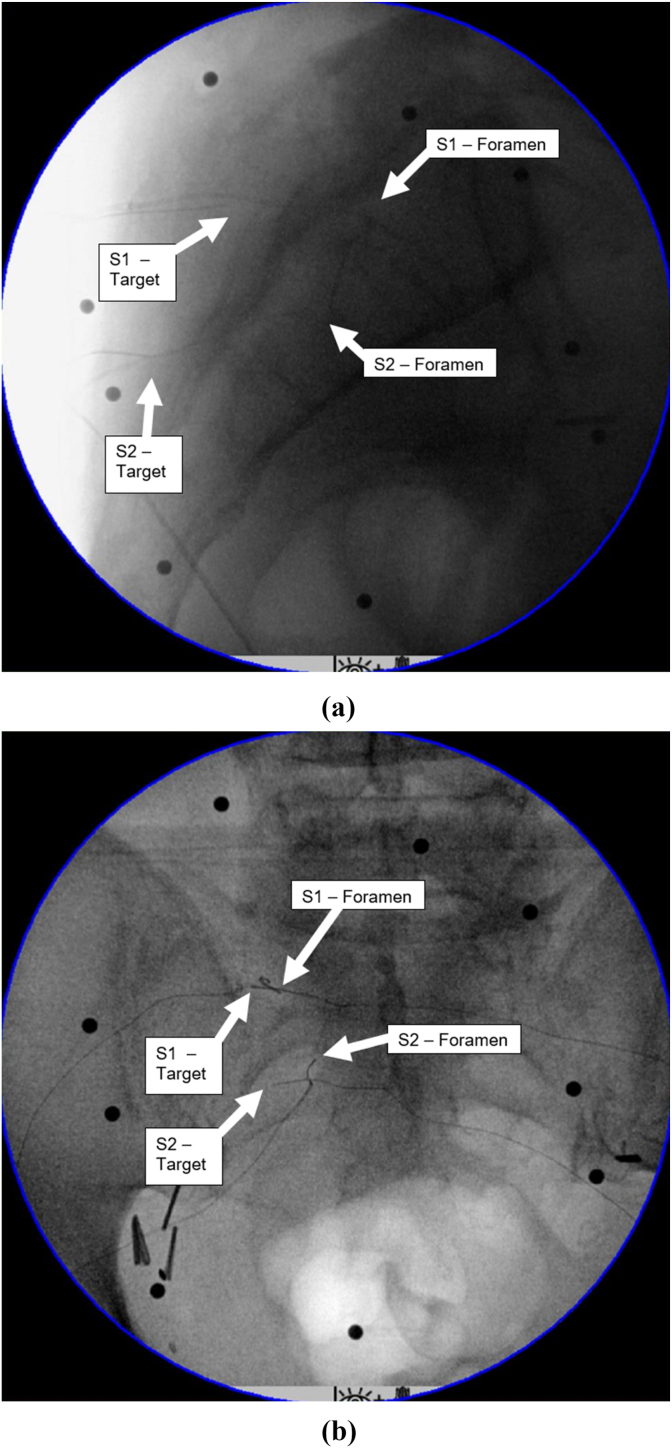
Labeled lateral (A) and posterior-to-anterior (B) x-rays of thermocouples post-implantation.

Under fluoroscopic guidance, a board-certified interventional pain physician used an OEC 9900 C-arm (General Electric Company, Boston, MA, USA) and 18G radiofrequency cannulas (Avanos Medical Inc, Alpharetta, GA, USA) to insert 30G k-Type thermocouples (Omega Engineering Inc., Norwalk, CT, USA). In the first cadaver, the thermocouples were positioned at four of the typical RFA targets for SIJ pain (L5 medial branch nerve and S1-S3 lateral aspect of the foramen). In the second cadaver, the thermocouples were placed at the same lateral targets for S1 and S2 and two additional thermocouples were placed inside the S1 and S2 foramen at the location of the nerve root (Fig. 1).

In both cadavers, the locations of the thermocouples were sonicated with the Neurolyser XR (FUSMobile Inc, Alpharetta, GA, USA) using 1000J or 2000J. The time-temperature history of each thermocouple was recorded using a digital thermocouple datalogger (PICO Technology, Cambridgeshire, UK). Adequate cooling time elapsed between sonications for the tissue to cool to a steady-state temperature. Finally, the heating ratios of the change in temperature recorded at the target and foraminal thermocouples were calculated as:

$$Heating\;Ratio\;\left(\Delta T\;Ratio\right)=\frac{\left(T_{Finish,Target}-T_{Start,Target}\right)}{\left(T_{Finish,Foramen}-T_{Start,Foramen}\right)}$$

### 2.2 *In silico* simulations

*k-Wave* is a MATLAB toolbox for simulating acoustic waves of intensity, geometric, frequency, and phase characteristics in arbitrary media. It solves a generalized Westervelt equation using a pseudo-spectral method [25]. *k-Wave* also implements Penne's bioheat equation, which uses the output of the acoustic simulation as an input and produces a time-domain thermal result.

A *k-Wave*- and CT-based simulation pipeline was used to acoustically and thermally simulate in an auto-segmented quasi-heterogeneous CT medium. Materials included in the simulation were gel pads (Parker Aquaflex, Paker Laboratories INC, Fairfield, NJ, USA), muscle, and bone. The simulation package has been described in previous publications [21,26]. The Graphics Processing Unit (GPU) accelerated code was run using an Nvidia Quadro RTX 8000 with 48 GB of video memory.

Twelve anonymized CT scans (SegMed, Palo Alto, CA, USA) were evaluated for fitness of SIJ simulation with the Neurolyser XR device. Selection criteria for the CT scans were:

• Wide field-of-view with no collimation where the skin line can be seen in the field of view, such that the target depth (distance between the target and skin on the central axis of the HIFU beam) is evaluable.
• CT scan in the axial dimension extending from at least the superior endplate of the L2 through the tip of the coccyx.
• <2 mm voxel size on the largest dimension of the voxel to minimize partial volume averaging when rescaling for *k-Wave* acoustic simulation.
• Head-first supine (HFS) positioning for CT scanning. (Although head-first prone is typical for treatment, it is exceedingly rare for patients to undergo CT scanning in that position, hence the choice of HFS).

Two scans were rejected because the targets were too deep (>75 mm to the skin) to treat with the Neurolyser XR. For the remaining 10 scans, the 3D DICOM central axis coordinates of the target and device were selected manually as input to the simulation. Using these two coordinates to define the line of the central axis of the HIFU device, the incident angle of the HIFU beams was 5° contralateral oblique and 10° cranial relative to CT geometry.

The *k-Wave* simulation volume then was delineated by the specified coordinates and based on the geometry of the Neurolyser XR transducer. The acoustic simulation was then run and the output Root Mean Square (RMS) pressure from that simulation was used as input into the *k-Wave* bioheat simulation. First, 300 J (J) were delivered as a non-ablative, sensory test at 15 acoustic Watts (W_Acoustic_). Then the 1000J ablations were delivered at 20 W_Acoustic_ at the same target before simulating the subsequent target in the same fashion. 300J and 1000J were chosen to simulate clinical energies used in previous works with the Neurolyser XR device [[19], [20], [21]] and planned energies for upcoming SIJ XRgHIFU human trials [23].

The cooling time allowed between sonications affects the resultant lesion because the thermal rise builds up if the temperature does not return to baseline before the next sonication. In clinical practice, it takes physicians about 30 s to 3 min to target the Neurolyser XR device, dependent on device familiarity and anatomical complexity. Because the purpose of this paper is to explore the safety of this treatment for SIJ, the worst-case scenario was considered: No cooling time between 300J and 1000J sonications was simulated and only 60 s of cooling time were simulated between the end of the 1000J sonication for one lesion and the 300J sonication for the next target. This scenario corresponds to the most conservative approach.

**Fig. 2:**
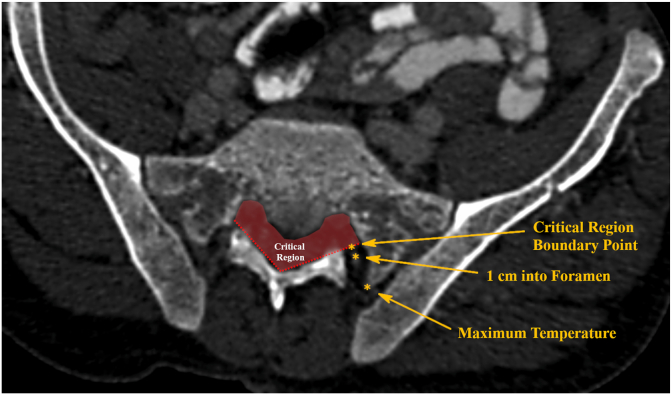
Diagram showing in-silico model S1 test points for Patient 68921. The boundary of the critical region is defined by the diagonal line corresponding to the anterior aspect of the lamina.

In the simulation, ablation volume is determined to occur anywhere the thermal dose reaches 240 cumulative equivalent minutes at 43 °C (CEM43) [27]. Temperatures immediately following sonication and CEM 43 test points (Fig. 2) were sampled at the maximum temperature location for each sonication, 1 cm into the S1 and S2 foramen, and a depth corresponding to the boundary of what would be the critical region for patients. The 1-cm and maximum temperature sample points mirrored the method used for the cadaveric study and the ratios were calculated in the same fashion. The critical region point was determined to be the diagonal line defined by the anterior margin of the lamina, because any ablation extending into that region may pose a risk to the nerve root/cauda equina. All S1 and S2 lesions were analyzed to determine whether a lesion occurred at this critical region boundary point, and a cumulative graph of lesion width was generated using all simulations.

The S1 targets of the 10 CT scans were then re-simulated with the same sonication and cooling times as the cadaveric model with perfusion set to zero to generate a time-temperature history comparable to that for the cadaveric model. These results were then normalized to the 300J sonication and compared with the thermocouple-measured thermal rise in the cadaveric model after being normalized with the same method.

## 3 Results

The ratio of the target thermocouple and the foraminal thermocouple to the ratio of the simulation hotspot and the sample point 1 cm into the foramen showed good agreement (Table 1). The cadaveric model ratio was 15.7 ± 4.6 (N = 6), whereas the simulation ratio was 17.3 ± 9.3 (N = 20). The ΔT ratio at the critical region boundary point was 24.5 ± 3.2 (N = 20).

**Table 1:** Change In Temperature Ratios Between the Hotspot/Target Thermocouple and the Sampled Foramen Point/Foraminal Thermocouple. Parentheticals denote the number of obselsjour-640ervations.

ΔT ratios at 1 cm test point/thermocouple location			
	All ratios	S1 ratios	S2 ratios
Cadaver	15.7 ± 4.6 (6)	18.2 ± 5.9 (2)	14.4 ± 3.3 (4)
*in silico*	17.3 ± 9.3 (20)	12.0 ± 5.3 (10)	22.5 ± 9.4 (10)

**Fig. 3:**
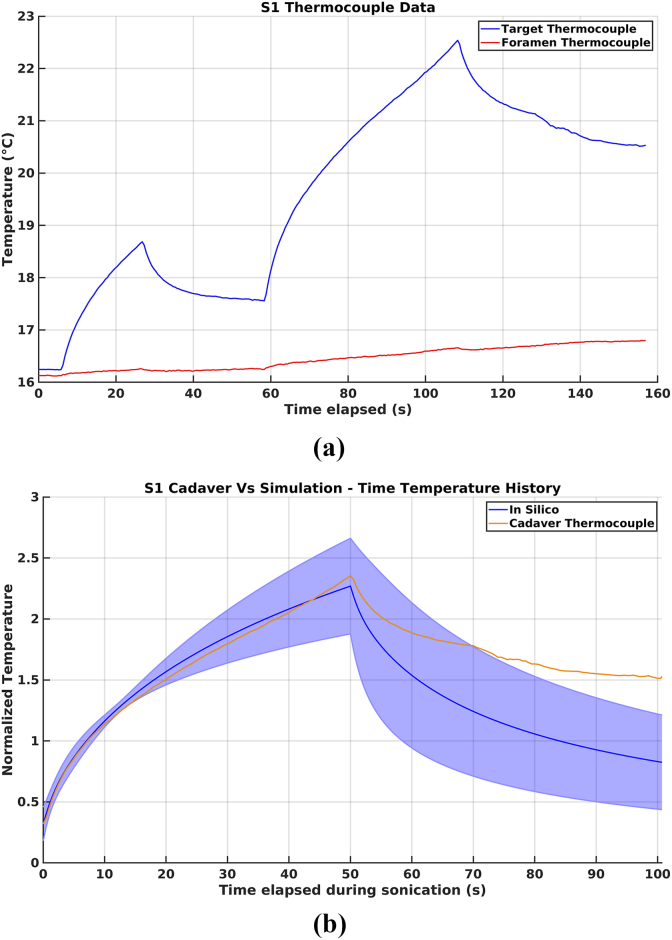
(A) Heating from S1 300J test and 1000 J treatment sonication. Blue is the S1 target thermocouple and red is the S1 foraminal thermocouple. (B) Time-temperature history from 1000J treatment sonication. Data are normalized to the heating achieved in the 300J test sonication for simulation and measurement, respectively. Blue indicates simulations in which the line is the average and the shaded region is the standard deviation and orange indicates the S1 target thermocouple measurement. (For interpretation of the references to colour in this figure legend, the reader is referred to the Web version of this article.)

The cadaveric model safety study showed little heating inside the foramen relative to the thermal rise achieved at the target thermocouple (Fig. 3A).

The time-temperature history comparison between *in silico* simulations showed agreement with the cadaveric model, although the cadaveric model cooled more slowly than the *in silico* model (Fig. 3B).

**Fig. 4:**
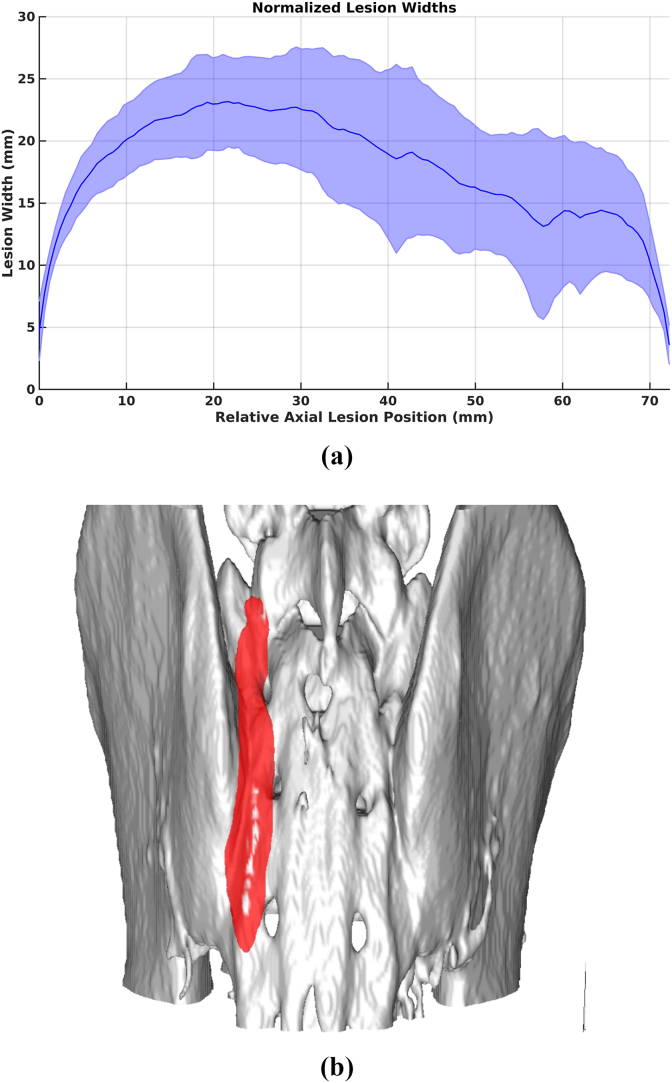
(A) Lesion widths for all patients simulated. Lesion volume is sectioned into axial slices then measured for each slice, moving from the foot toward the head (HFS DICOM orientation position) and scaled such that every lesion is the same length. Zero represents the inferior-most axial slice on which a lesion forms. B) Example of composite strip lesion (patient 45858 k-Wave simulation).

The *in-silico* model indicated that a continuous strip lesion was achieved in all patients (Fig. 4), and the lesions were wide enough to anticipate a robust therapeutic result. No simulations showed ablation at the critical region boundary point. The ΔT ratio observed for the critical region boundary point when compared with the maximum temperature was 24.5 ± 3.2 (N = 20).

## 4 Discussion

This study supports the safety of SIJ HIFU with the Neurolyser XR. The heating ratio in both the cadaveric and *in-silico* models agree and demonstrate safe heating of critical structures within the foramen for treatments simulated. In the cadaveric model, sonicating thermocouples placed at the target locations demonstrated that the physician was reliably able to target relevant treatment locations to treat sacroiliitis and avoid the foramen. The *in-silico* model produced a contiguous strip lesion using a seven-target method on every patient simulated. The average width of the *in-silico* strip lesion was ≥15 mm with >95% of the lesions generated being significantly larger and wider than comparable conventional and cooled RFA lesions [28].

### 4.1 Comparison between models

The agreement between the cadaver and *in-silico* models supports the safety of SIJ HIFU with the Neurolyser XR because the heating ratio suggests safe heating of critical structures. Using the average heating ratio of 24.5 between the critical region boundary point and the maximum temperature achieved in the volume, every 1 °C of thermal rise at the target produced, on average, a ∼0.042 °C thermal rise at the critical region boundary point. Boiling caps the maximum temperature achievable in tissue to 100 °C and the initial temperature can be approximated at 37 °C, which would amount to a thermal rise of 63 °C in the target and 2.6 °C of thermal rise at the boundary of the critical region. Even under these extreme and conservative assumptions, a maximum tissue temperature of 39.6 °C at the critical region boundary point is well below the 43 °C temperature threshold for thermal ablation.

ΔT ratio was chosen as the primary comparison between the cadaveric and *in-silico* models because of tissue properties. Although acoustic characteristics are preserved better in salt cure-embalmed than in formalin-fixed cadavers, tissue does degrade and is not exactly equivalent to living tissue. Conversely, the *in-silico* tissue model is pseudo-heterogenous, meaning that the only tissues segmented are cortical bone and muscle, ignoring fat, microcalcifications, and other tissue heterogeneities that might impact acoustic and thermal results.

Simplifying the analysis to a ratio normalizes the differing thermal and acoustic tissue properties between the two models and the differing device parameters used, such as the energy delivered. This supports the validity of the *in-silico* model, which contains significantly more information than thermal point measurements provided by a thermocouple in the cadaveric model. Further, this ratio has some clinical relevance, because the nerve root/cauda equina is the most relevant critical structure for future SIJ HIFU treatment, and it can be accessed in soft tissue through the foramen.

Analyzing the agreement between the two models when comparing the average S1 *in-silico* versus cadaver and S2 *in-silico* versus cadaver, the ΔT ratio increased comparing S1 to S2 *in silico* and the ΔT decreased comparing S1 to S2 in the cadaver. These observations agree with each other in the context of the small sample size and study error. Analyzing at the average, this trend may be observed for a variety of reasons, such as the specific anatomy of this cadaver compared with the CT scans used and variations in placement depth of the thermocouple relative to the depth of 1 cm used in the *in-silico* model.

The comparison of time-temperature histories between the simulation and the second cadaver's foramen and target thermocouples showed similar behavior, with a few caveats. First, perfusion was set to zero in the *k-Wave* simulation to mimic the cadaver. Even with perfusion set to zero, the cadaver showed slower cooling relative to the simulation, indicating that there was a difference between the thermal conductivity assumed by the simulation and the cadaveric model/thermocouple system. The *in-silico* model without perfusion also showed more heating in the initial test 300J sonication than the cadaveric model. Without knowing the exact tissue properties of the cadaver, it is difficult to identify the source of the discrepancies between the two models in time-temperature histories, but it most likely is due to different material properties between what is assumed by the simulation and the cadaver.

### 4.2 Cadaveric model

In the first cadaver, sonicating thermocouples placed at the target locations for SIJ treatments showed that the physician was reliably able to target relevant treatment locations to treat sacroiliitis. Clinical insight was also gained in simulating a SIJ HIFU treatment on a cadaver, demonstrating which cranio-caudal and oblique angles were necessary to achieve the desired clinical result while avoiding the posterior superior iliac spine and superior articular process. Although these angles are patient-dependent, a good heuristic of ∼5° cranial and ∼10° contralateral oblique relative to the anatomy was established. In terms of target depth and location, these results are somewhat generalizable to the wider sacroiliitis patient population, the target depths and locations fall squarely in the ranges described in previous work, per the lateral view shown [29].

Results of the foramen safety test on the second cadaver showed strong consistency across all six sonications, which supports the safety of this procedure for treatment of SIJ pain. Although the data are limited to a single female cadaver and only a few sonications, they provide preliminary evidence of procedure safety.

### 4.3 *In-silico* model

A contiguous strip lesion was seen, using a seven-target method on every patient simulated. The average width of the lesion was ≥15 mm with >95% of the lesions generated being significantly larger and wider than comparable conventional and cooled RFA lesions [28]. This continuous strip lesion is evident regardless of patient habitus. Although lesions were narrower on superior targets, the lesion volume delineated by the *k-Wave* simulations show the strip lesion produced appears to significantly overlap with the lateral sacral branches [30]. The large widths of the strip lesion (≥15 mm on average) suggest robust ablation of the lateral sacral branches. Further, minimal penetration into the foramen suggested a high degree of safety.

### 4.4 Limitations

As previously discussed, the differences between tissue properties between the cadaver and the simulation models are a limitation of the study, though this is compensated for by having the heating ratio as the primary endpoint. A lack of perfusion and thermoregulation limits the generalizability to *in vivo* tissues. Further, omitting perfusion in the *in-silico* model leads to a slight overestimation of the lesion size and volume, while in the cadaver model the effect of no perfusion is likely dominated by the differences between material properties of the preserved tissue when compared to *in vivo* human tissue.

Sample size is another limitation of this study. A single female cadaver was used and only 10 of 12 CT datasets were used as two of the patients in the initially acquired 12 datasets from SegMed Inc. were not simulated because the skin-to-target distance in them was greater than 75 mm, which is deeper than the Neurolyser XR can treat.

Limitations of the *in-silico* model include that it assigns idealized material properties, ignoring confounding tissue heterogeneity. Thus, simulation tends to slightly over-predict lesion volume. Further, all CT scans used were scanned from a HFS position, which is not the treatment position for this procedure. The conventional patient position for this treatment with RFA is prone with pillows under the abdomen, which pushes the superior aspect of the sacrum posterior as it flattens the back. As such, the CT scans overestimate the depth of superior targets. Tissue attenuation and incoherent focal spot placement can underestimate lesion volume, although that is likely more than offset by overestimation due to idealized material properties. Finally, the cadaveric version of the *k-Wave* simulations with perfusion set to zero does not account for acoustic or thermal perturbances from the 30G implanted metal thermocouples.

Finally, all authors except LK have significant conflicts of interest. While efforts were made to mitigate bias by making the safety endpoints of the study principally quantitative in nature and offering up supplemental data upon request, the potential bias in study design and interpretation due to the conflict of interest of the authors cannot be ignored.

## 5 Conclusions

The present combined cadaveric and simulation study provides preliminary data suggesting that using the Neurolyser XR for XRgHIFU ablation of the lateral branches to treat sacroiliitis is safe and feasible. The procedure will produce a large contiguous strip lesion 10-25 mm in diameter that is likely to include all posterior sacral lateral branches. This lesion is unlikely to pose a risk of ablation of critical structures such as the nerve root/cauda equina within the foramen. The data from the two models support the safety and feasibility of using the Neurolyser XR to treat pain from sacroiliitis. Further work should include animal and/or preliminary clinical studies to further confirm these results.

## Patient consent for publication

Not applicable.

## Contributors

LK, EM, AH, and BS designed and LK performed the cadaveric portion of the study. EM, J-FA, AH, MG, and SL designed the *in-silico* portion of the study, EM wrote the simulation package and performed the simulations, and EM, J-FA, AH, MG, SL, and LK analyzed the *in-silico* results. All authors participated in writing the manuscript.

## Ethics approval

Not applicable.

## Provenance and peer review

Not commissioned; externally peer reviewed.

## Data availability statement

Supplemental data are available upon request.

## Open access

This is an open access article distributed in accordance with the Creative Commons Attribution Non-Commercial (CC BY-NC 4.0) license, which permits others to distribute, remix, adapt, build upon this work non-commercially, and license their derivative works on different terms, provided the original work is properly cited, an indication of whether changes were made, and the use is non-commercial. See: http://creativecommons.org/licenses/by-nc/4.0/.

## Funding

Financial support was provided by FUSMobile to cover the research costs of both the cadaveric and *in-silico* portions of this study.
